# Blocking CGRP in migraine patients – a review of pros and cons

**DOI:** 10.1186/s10194-017-0807-1

**Published:** 2017-09-25

**Authors:** Marie Deen, Edvige Correnti, Katharina Kamm, Tim Kelderman, Laura Papetti, Eloisa Rubio-Beltrán, Simone Vigneri, Lars Edvinsson, Antoinette Maassen Van Den Brink, On behalf of the European Headache Federation School of Advanced Studies (EHF-SAS)

**Affiliations:** 1 Danish Headache Center, Department of Neurology, Rigshospitalet, Copenhagen, Denmark; 2 Department of Child Neuropsychiatry, University of Palermo, Palermo, Italy; 3 Department of Neurology, University Hospital, LMU, Munich, Germany; 4 Department of Neurology, Ghent University Hospital, Ghent, Belgium; 5 Headache Center, Bambino Gesù Children’s Hospital, IRCCS, Rome, Italy; 6 Division of Vascular Medicine and Pharmacology, Department of Internal Medicine, Erasmus University Medical Center, Rotterdam, The Netherlands; 7 Department of Experimental Biomedicine and Clinical Neurosciences, University of Palermo; Advanced Algology Research and Pain Medicine Unit, Santa Maria Maddalena Hospital, Occhiobello, Italy; 8 Department of Internal Medicine, Institute of Clinical Sciences, Lund University, Lund, Sweden

**Keywords:** Migraine, Cgrp, Cgrp receptor, Prophylactic treatment, Acute treatment, Gepants

## Abstract

Migraine is the most prevalent neurological disorder worldwide and it has immense socioeconomic impact. Currently, preventative treatment options for migraine include drugs developed for diseases other than migraine such as hypertension, depression and epilepsy. During the last decade, however, blocking calcitonin gene-related peptide (CGRP) has emerged as a possible mechanism for prevention of migraine attacks. CGRP has been shown to be released during migraine attacks and it may play a causative role in induction of migraine attacks. Here, we review the pros and cons of blocking CGRP in migraine patients. To date, two different classes of drugs blocking CGRP have been developed: small molecule CGRP receptor antagonists (gepants), and monoclonal antibodies, targeting either CGRP or the CGRP receptor. Several trials have been conducted to test the efficacy and safety of these drugs. In general, a superior efficacy compared to placebo has been shown, especially with regards to the antibodies. In addition, the efficacy is in line with other currently used prophylactic treatments. The drugs have also been well tolerated, except for some of the gepants, which induced a transient increase in transaminases. Thus, blocking CGRP in migraine patients is seemingly both efficient and well tolerated. However, CGRP and its receptor are abundantly present in both the vasculature, and in the peripheral and central nervous system, and are involved in several physiological processes. Therefore, blocking CGRP may pose a risk in subjects with comorbidities such as cardiovascular diseases. In addition, long-term effects are still unknown. Evidence from animal studies suggests that blocking CGRP may induce constipation, affect the homeostatic functions of the pituitary hormones or attenuate wound healing. However, these effects have so far not been reported in human studies. In conclusion, this review suggests that, based on current knowledge, the pros of blocking CGRP in migraine patients exceed the cons.

## Review

Migraine is a highly prevalent and disabling disorder for which treatment options are still inadequate. The underlying pathophysiology is largely unknown, but calcitonin gene-related peptide (CGRP) most likely plays an important role. The first time CGRP was hypothesized to be involved in migraine was in 1985 [1]. This hypothesis was later supported by the finding of CGRP release during acute migraine attacks and the subsequent demonstration of normalization of CGRP levels in migraine patients after efficacious sumatriptan treatment [2]. In animal studies, triptans also inhibit the release of CGRP [3]. Evidence for a causative role of CGRP in migraine came from a study showing that intravenous provocation with CGRP induces migraine-like attacks in migraine patients [4]. This led to focus on this peptide and its receptor as a possible target for new migraine therapies.

CGRP and its receptor are expressed in both the peripheral and the central nervous system (CNS), including the trigeminovascular pathways. More than 30 years ago CGRP was demonstrated in trigeminal ganglion (TG) pseudounipolar neurons [5]. These neurons connect cranial structures to the central nervous system at the lower brainstem, caudal part of the trigeminal nucleus caudalis and upper spinal cord at C1-C2 [6]. In the peripheral trigeminovascular system, as well as in the TG, CGRP is located in about 50% of the neurons and in unmyelinated C­fibers, whereas the CGRP receptor elements are expressed in about 40% of the TG neurons and in myelinated A-fibers, which connect the PNS with the CNS [7, 8]. In humans, CGRP is present in two isoforms, α-CGRP and β-CGRP, where α-CGRP is most abundantly found in primary spinal afferents from sensory ganglia, whereas β-CGRP is mainly found in the enteric nervous system [6]. The CGRP receptor consists of three subunits: receptor activity-modifying protein 1 (RAMP1), calcitonin-like receptor (CLR) and receptor component protein (RCP) [9]. As well as playing a role in cranial nociception [10], CGRP is a potent general arterial vasodilator. At peripheral synapses, CGRP released from trigeminal terminals results in vasodilation via CGRP receptors on the smooth muscle cells of meningeal and cerebral blood vessels [8, 11]. CGRP and its receptor are also located in the cardiovascular system where they are assumed to exert a protective role [9, 12].

The first designer drug able to competitively block the effect of CGRP was olcegepant [13]. This nonpeptide CGRP-receptor antagonist showed high efficacy but had a low oral bioavailability [14]. This led, however, to the synthesis of several other small molecule CGRP receptor antagonists. This class was later called the gepants. Though promising with regards to efficacy, further development of some of the gepants was discontinued due to liver toxicity upon repeated exposure [15]. Encouraged by the efficacy of blocking CGRP for the treatment of migraine, monoclonal antibodies able to block either CGRP or its receptor were developed and tested in several preclinical modalities [16, 17]. The antibodies are designer drugs that are highly specific for the target but about 500 times the size of gepants or triptans [6]. They have been designed for prophylactic use in frequent episodic and chronic migraine. In this review, we will discuss the pros and cons of blocking CGRP in migraine patients. We will review the efficacy and safety of already tested drugs and compare it to the efficacy and safety of topiramate, a widely-used migraine prophylactic. Additionally, we will review the possible consequences of blocking CGRP based on findings from animal studies. Lastly, we will discuss other concerns such as long-term use and cost of the treatment.

### Efficacy of CGRP (receptor) blockade: Evidence from double-blind, placebo-controlled trials

**Fig. 1:**
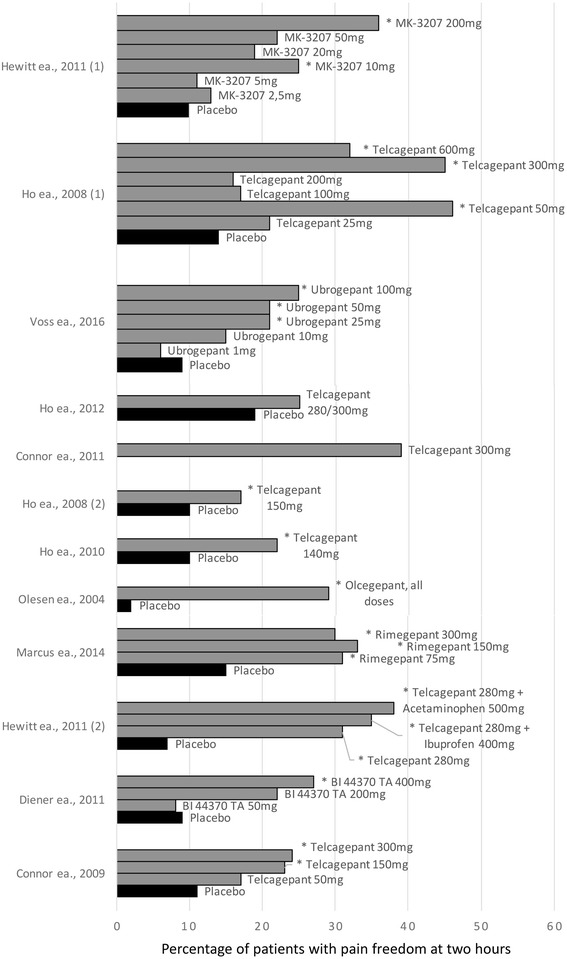
Efficacy of gepants in the acute treatment of migraine. Bars indicated with * represents statistically significant values compared to placebo (*p* < 0.05)

In 2004, a proof-of-concept study showed that intravenous olcegepant was effective in the acute treatment of migraine [18]. Since then, five other gepants have been tested for the acute treatment of migraine [19–33]. Figure 1 provides an overview of the efficacy data for these agents. All gepants were significantly better than placebo at achieving their primary outcome at adequate doses: pain freedom or relief at 2 h. Only one study, a study on safety in coronary patients, could not demonstrate difference in pain freedom at 2 h after telcagepant; however, only 165 of the planned 400 patients were included, reducing the statistical power of this study [27].

Five of these studies also included a comparison to a triptan [19, 21, 26, 29, 30]. In one of these studies, telcagepant showed a numerically higher efficacy than rizatriptan with regards to sustained pain relief [29]. In other trials, the efficacy of telcagepant, BI44370 and rimegepant showed no significant difference to that of zolmitriptan (5 mg), eletriptan (40 mg) and sumatriptan (100 mg), respectively [21, 26, 30]. In one large study, assessing the long-term safety of telcagepant, 19,820 attacks were treated with telcagepant and 10,981 attacks with rizatriptan. For two endpoints, pain freedom and pain relief at 2 h, rizatriptan was superior compared to telcagepant (OR <1 in favor of rizatriptan. OR (95% CI): 0.58 (0.45, 0.75) and 0.70 (0.55, 0.89), respectively). For all other pre-specified efficacy outcome measurements, no difference was found between the efficacy of telcagepant and rizatriptan at 2 h [19].

Telcagepant has also been tested as prophylactic treatment of episodic migraine [25, 28]. The first of these studies was terminated early due to adverse events and the pre-specified analyses could not be performed. However, post-hoc analysis showed telcagepant to be effective at four weeks in reducing migraine days [25]. In the second study, in a population of patients with perimenstrual migraine, administration of telcagepant in the perimenstrual period did not result in a significant reduction in mean monthly headache days, which was the primary endpoint [28]. There was a reduction of mean monthly on-drug headache days, but the reliability of this analysis is questionable, since no correction for multiple comparisons was done.

**Fig. 2:**
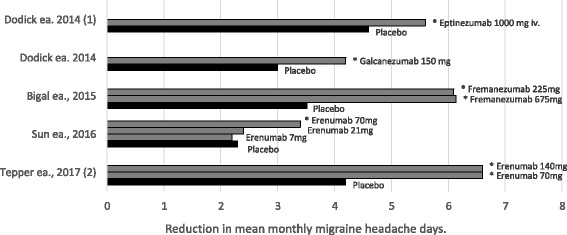
Efficacy of monoclonal antibodies in the preventive treatment of migraine. Bars indicated with * represents statistically significant values compared to placebo (*p* < 0.05). (1) had change in mean monthly migraine days from baseline to weeks 5–8 as the primary endpoint. All other studies had change in monthly migraine days from baseline to weeks 9–12 of the 12-week double-blind treatment phase as the primary endpoint. In (1) the drug/placebo was administered intravenously. In all other studies, the drug/placebo were given subcutaneously. (2) is on chronic migraine patients. All other studies are on episodic migraine patients

Antibodies against CGRP or the CGRP receptor have been tested as prophylactic treatment of episodic and chronic migraine. To date, four agents have been studied in six clinical studies [31, 32, 34–37]. Figure 2 provides an overview of the efficacy data of the studies where reduction in migraine days was the primary endpoint. All monoclonal antibodies showed a significant reduction in their primary endpoint, either mean change from baseline in monthly migraine days (5 studies) or mean change in headache hours from baseline (1 study). These agents had an additional reduction over placebo of between 1 and 2.8 migraine days per month (when not considering the inefficacious lower doses of erenumab). In the study in chronic migraine, where change in headache hours was the primary outcome (data not included in the figure), the additional reduction over placebo was 22.7 and 30.4 h per month for the two doses tested [37].

Interestingly, in one study, 11 of the 67 (16%) patients who had 5 to 14 migraine days per month at baseline, experienced no migraine days during the 12 week study period, versus no patients in the placebo group [34]. In another study, 31 of 98 (32%) patients reporting 4 to 15 migraine days at baseline had a 100% response (defined as a 28-day migraine free period over the 12-week treatment period). In the placebo group, only 18 of 104 (17%) of placebo patients had a 100% response [35]. No other studies reported on the 100% responder rate. Although these data seem interesting, they come from post-hoc analyses and so their significance remains unclear.

The data from these 20 studies provides robust and consistent evidence for a crucial role of CGRP in migraine pathophysiology and a high efficacy of blocking CGRP as a prophylactic treatment.

### Is blocking CGRP as or more efficient than current preventative treatments?

Current preventative treatment options for migraine include antihypertensive drugs, antidepressants and antiepileptic medication. In contrast to CGRP (receptor) blockers, these have all been developed for diseases other than migraine and it is estimated that less than 50% of patients on prophylactics experience a 50% reduction in their monthly attack frequency [38].

Topiramate was proven efficient as a preventative treatment of episodic migraine after positive results from three randomized, multi-center, placebo-controlled studies. Thus, topiramate is currently recommended as a level A medication for prevention of episodic migraine with established efficacy (≥ 2 Class I trials) in the 2012 AHS/AAN guidelines [39]. Here, we review so far published data from Phase III studies of the monoclonal antibodies [40–43] in relation to pivotal studies on topiramate [44–48] in episodic and chronic migraine.

In three phase III studies, including over 1500 patients, topiramate 100 mg/d significantly reduced the number of monthly migraine days compared to placebo (reduction of monthly migraine days about −1.8 to −2.6 for topiramate vs. -1.0 to −1.3 for placebo). The ≥50% responder rates were also significantly higher for topiramate (37–54% vs. 22–23%, respectively) [44–46]. In the so far available data from Phase III studies of CGRP (receptor) antibodies, blocking of CGRP showed a similar efficacy with a reduction of monthly migraine days from baseline of −2.9 (verum) vs. -1.8 (placebo) for erenumab (AMG-334) [43]; −4.3 (300 mg)/−3.9(100 mg) vs. -3.2 (placebo) for eptinezumab (ALD-403), [41]; −4.0 (120 mg)/−3.8 (240 mg) vs. -2.15 (placebo) for galcanezumab (LY2951742) [40] and −3.7 (225 mg monthly)/−3.4 (675 mg quarterly) vs. -2.2 (placebo) for fremanezumab (TEV-48125) [42]. The ≥50% responder rates were also significantly higher than for placebo and similar, albeit a little higher, to those of topiramate, ranging from 56.3% to 62.3% (≥50% responder rates: eptinezumab: 56.3% (300 mg)/ 49.8% (100 mg) vs. 37.4% (placebo) [41]; galcanezumab: 62.3% (120 mg)/ 60.9% (120 mg) vs. 38.6% (placebo) [40]).

Topiramate has also proven efficacious in patients with chronic migraine [47, 48]. In two randomized, placebo-controlled, double-blinded studies with 387 subjects with a daily dose of 100 mg or 50-200 mg topiramate showed a significant reduction in monthly migraine days compared to placebo (−6.4 (±5.8) vs. -4.7 (±6.1) [47] and −3.5 (±6.3) vs. +0.2 (±4.7) [48]). The ≥50% responder rate was also significantly higher for topiramate (22% vs. 0% for placebo) [48]. In line with this, blocking of CGRP significantly reduced the number of monthly headache migraine days in 1113 chronic migraine patients with an average of 19.4 headache days (−4.8 (120 mg)/−4.6 (240 mg) vs. -2.7 (placebo)). Likewise, the ≥50% responder rate was significantly higher for the active drug compared to placebo. (27.6% (120 mg)/ 27.5% (240 mg) vs. 15.4%) [49].

Another important aspect of medication is the incidence and severity of adverse events. Compared to topiramate, adverse events reported from CGRP trials were generally mild and less frequent. Upper respiratory tract infection/nasopharyngitis, and injection-site pain have so far been the most frequent reported adverse events [40–43] (see next paragraph for more details). In contrast, reported adverse events of topiramate, such as taste disturbance, weight loss, anorexia, fatigue, memory problems and paresthesia were more common in the active groups than in the placebo groups.

## Safety issues regarding blocking of CGRP – Are there any?

### Evidence from clinical studies

Even though the knowledge of the presence and function of CGRP in the CNS is sparse, the function in both the peripheral and enteric nervous system is well established and CGRP is expressed widely throughout both systems. Thus, a wide variety of possible adverse events could be anticipated when blocking CGRP. However, reported adverse events after blocking of CGRP have in general been mild to moderate and the incidences have been low.

Among the first CGRP receptor antagonists under trial, intravenous olcegepant caused mild to moderate adverse events such as paresthesia, nausea, headache, dry mouth and unspecific vision disturbances in a minority of patients [18]. However, more serious adverse events were reported with telcagepant and MK-3207, which caused liver toxicity with transient increase of transaminases in a small group of included subjects (*n* = 13 for telcagepant) upon repeated doses. This lead to discontinuation of the trial program for these molecules [15, 25]. Other non-peptide CGRP receptor antagonists such as BI44370TA, BMS-927711, and, most recently, MK-1602 have also been tested. For all three molecules adverse events were mild to moderate and the incidence was low and similar to the placebo group [21, 30, 33]. No liver toxicity was reported for these drugs, and the gepant program is thus still ongoing.

More recently, great attention has been given to the development and testing of monoclonal antibodies (mABs) targeting circulating CGRP or its receptors. Most importantly, none of these drugs show liver toxicity. This is in line with the theoretical probability of mABs causing liver toxicity, which is very low, since metabolism of mABs do not result in production of toxic metabolites [50]. In addition, despite the potentially harmful inhibition of vasodilation due to CGRP inhibition, no cardiovascular concerns have been disclosed with any of these drugs [51]. In trials, eptinezumab, galcanezumab and fremanezumab, monoclonal antibodies which all target CGRP, showed variable percentages of adverse events, which in line with the gepants, were mild to moderate (e.g. upper respiratory or urinary tract infection, fatigue, back pain, arthralgia, nausea and vomiting). Erenumab (AMG 334), which binds to the CGRP receptor, was also safe and well tolerated in a phase 2 trial [31].

In line with the poor chance of both the non-peptide CGRP receptor antagonists and the antibodies crossing the blood-brain barrier (BBB) [52], no central side effects have been reported so far. Therefore, although crossing of the BBB is likely to occur to some extent – telcagepant has been detected in primates cerebrospinal fluid, suggesting its presence in the CNS [53] – a central effect – and side effect – of these drugs seems unlikely.

### Do preclinical studies give reason to be concerned about side effects?

**Fig. 3:**
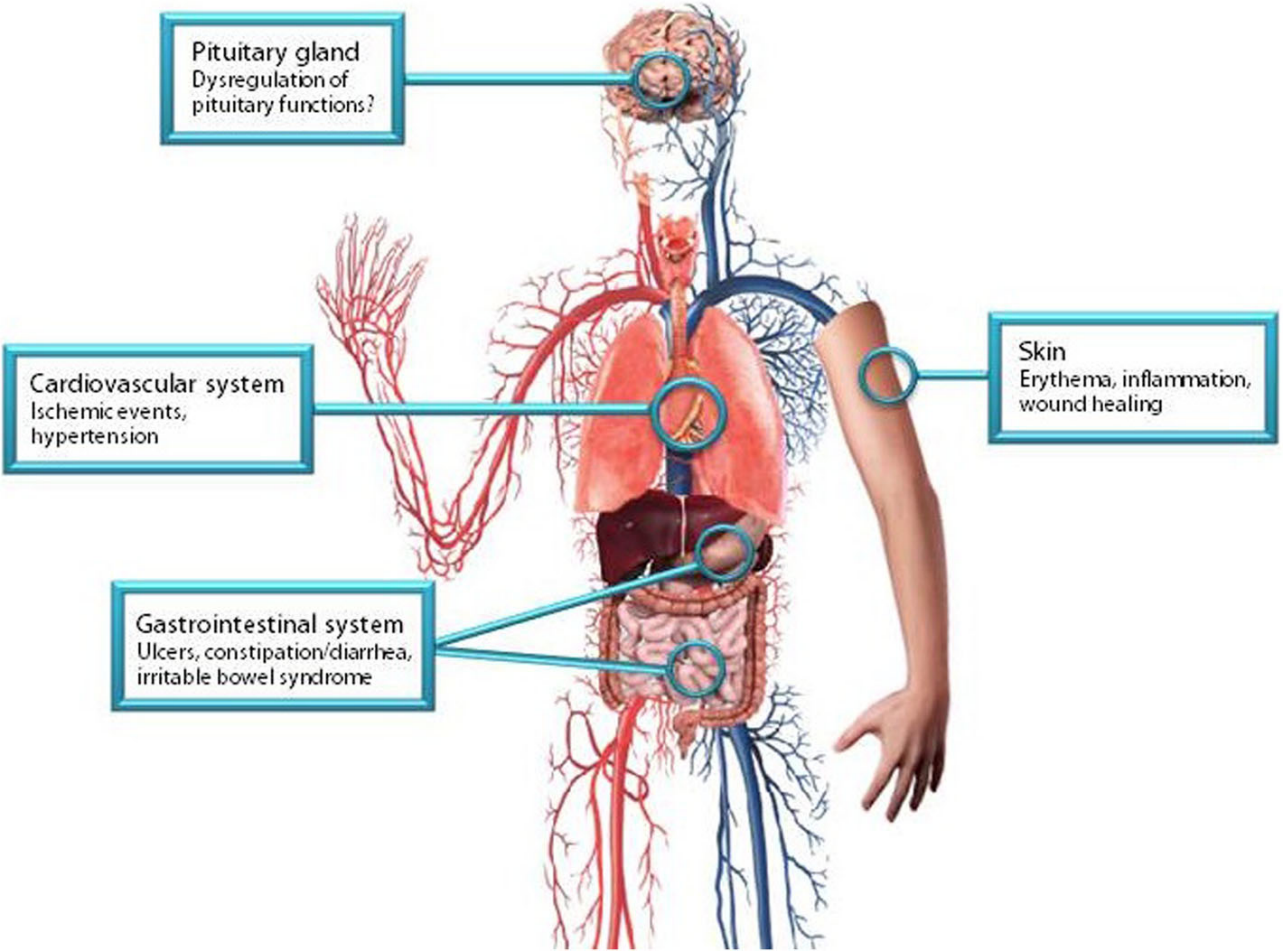
Possible side effects after long-term exposure to CGRP (receptor)-antibodies. An overview of the organ systems where CGRP and the receptor are present and possible side effects that could be caused by the non-selective blockade of α- and β-CGRP with the CGRP (receptor)-antibodies

CGRP is an ubiquitous peptide that is not only involved in migraine, but also in several physiological processes [12] and in homeostatic responses during pathophysiological conditions (Fig. 3) [9, 12]. As such, it is vital to consider the possible side effects caused by the non-selective blockade of α- and β-CGRP with the CGRP (receptor)-antibodies. As discussed in the previous section, the adverse events of the Phase II trials were mild [31, 32, 34–37], but it should be noted that the duration of these trials is not sufficient to see the long-term effects of continuingly blocking CGRP or its receptor.

In the cardiovascular system, CGRP is present in nerve fibers that innervate blood vessels [54] and the heart [55, 56], and participates in the regulation of blood pressure [12, 57–59]. Furthermore, it has also been described to have a role in the maintenance of (cardio)vascular homeostasis during ischemic events [9] and in tissue remodeling in pulmonary hypertension [60]. This protective role raises a concern, since migraine patients present an increased cardiovascular risk [61, 62]. This topic was recently reviewed elsewhere [9]. Hence, it is important to consider preexisting cardiovascular risk factors in patients (i.e. family history, tobacco exposure, obesity) to prevent a possible cardiovascular event.

Although CGRP participates in inflammatory processes [63–65], it has also been associated with facilitation of wound healing [66]. This is thought to be mediated through its ability to promote keratinocytes proliferation [67], enhance revascularization [68], reduce expression of tumor necrosis factor-α (TNF-α) and attenuate macrophage infiltration [69]. A consequence of blocking CGRP could thus be alterations in wound healing and increased inflammatory responses in skin injuries at the site of injection for the antibodies. However, this is a theoretical risk which has so far not been observed in clinical trials.

The antibodies against CGRP are not selective for α-CGRP but also block β-CGRP. The gastrointestinal tract is highly innervated by β-CGRPergic fibers from the enteric nervous system [70, 71]. In fact, animal studies with antibodies against CGRP showed extensive mucosal damage [72, 73], suggesting a role of CGRP in maintaining the mucosal integrity of the gastrointestinal tract. Blocking this could thus contribute to inflammatory bowel disease. Gastrointestinal motility is also considered to be modulated by CGRP, and administration of this peptide induces a dose-dependent biphasic response [74], which could lead to episodes of diarrhea or constipation. Furthermore, studies with CGRP KO mice have suggested CGRP agonists as a possible treatment for ulcer healing [75]; therefore, monitoring of gastrointestinal complications (i.e. ulcers, constipation) is recommended, even though 12 week studies have not reported these.

Finally, since it, as mentioned, is unlikely that the antibodies cross the BBB – and unlikely that the BBB penetration is changed during migraine attacks [76, 77] – it is important to consider the structures from CNS that are not protected by the BBB. Recent studies have demonstrated that the TG, together with the pituitary, are outside the BBB [78]. An effect on the TG could thus, partly, explain the therapeutic effect of the antibodies. However, CGRP and its receptor are also expressed in the anterior pituitary, suggesting a possible involvement in the regulation of hypothalamo-pituitary tract functions [79]. The exact involvement is still unknown, and further studies are needed to determine the long-term effects of blocking CGRP on the homeostatic functions of the pituitary hormones.

### Other considerations

Even though blocking of CGRP seems to be an efficacious and safe preventative treatment of migraine, there are many other aspects to consider with regards to the pros and cons of blocking CGRP in migraine patients.

Firstly, the administration of the newly developed monoclonal antibodies is either intravenous or subcutaneous. This could potentially cause complications at the injection site, and common adverse events in those treated with fremanezumab, galcanezumab and erenumab were indeed mild injection-site pain, pruritus and erythema [80]_._ A disadvantage of the intravenous administration route is the need of it being administered by a medical doctor. This not only increases the placebo response in clinical trials, but does also require for the patient to spend time visiting the clinic – increasing the risk of pathologization of the patient. However, the monthly administration, which is feasible due to the long half-lives of the medication, could improve adherence and compliance to medication, which is a common problem in treating migraine [81, 82]. Additionally, the CGRP antibodies seem to show a low risk for drug-drug interactions and hepatotoxicity since they are metabolized by degradation into peptides and single amino acids [83], which could be important for patients using multiple medications.

Secondly, as mentioned, the long-term risks of blocking CGRP are still unknown. Even though the absence of liver toxicity or other abnormalities in routine blood testing is in support of no or low long-term risks [80], studies testing the cardiovascular safety of the long-term blockade are warranted in order to answer the numerous questions on the possibility of higher risk in cardio- and cerebrovascular compromised patients. For example, it is unknown whether blocking CGRP could potentially transform transient mild cerebral ischemia into a full-blown brain infarct [9] and whether these risks are higher in women [9, 84]. To investigate these aspects, future studies should include patients with preexisting cardiovascular conditions.

Thirdly, the exact site of action of blocking CGRP is still partly unknown and CGRP could exert its effects on receptors distinct from the CGRP receptor [9]. Recently it was put forward that CGRP may act on the amylin receptor in TG [85] as well as in human coronary arteries [86]. If this is the case, this could pose an additional – unknown – potential risk of wiping out CGRP. We can also only guess whether patients not benefitting from receptor blockade would benefit from blockage of the peptide itself. Future studies should investigate how to differentiate responders from non-responders.

Lastly, a disadvantage when using antibodies is the risk of development of antibodies against the drug [15]. Indeed, antidrug antibodies were detected with all four antibodies [80], but these did not seem to affect efficacy [31]. However, long-term studies are needed to investigate whether, at long term, neutralizing antidrug antibodies will pose a problem for efficacy and safety of blocking CGRP with monoclonal antibodies. Finally, it is well known that antibody treatment is costly and the price of the drugs has to be taken into consideration when deciding whether to use CGRP antibodies as a prophylactic treatment and which patient groups to treat.

## Conclusion

Here, we have reviewed the pros and cons of blocking CGRP in migraine patients. In favor of using blocking of CGRP as a treatment of migraine, is that – based on evidence from clinical trials – whether using small molecule receptor antagonists or antibodies, the treatment is efficacious. Additionally, the liver toxicity induced by some of the gepants is not present with the antibodies, which are well tolerated. Lastly, in contrast to current prophylactic treatments, the drugs are developed specifically for migraine, based on findings from human migraine studies. Thus, the drugs may exert a more direct effect on migraine specific pathways than previously used prophylactic drugs. In addition, this provides hope and encouragement for further research into the pathophysiological mechanisms of migraine and potentially the discovery of other migraine specific therapeutic targets.

Speaking against chronically blocking CGRP, the long-term effects, particularly regarding the cardiovascular risks, are still unknown as well as the exact mode of action of the antibodies. In addition, development of neutralizing antidrug antibodies may, with time, affect the efficacy of the antibodies. Lastly, as with all antibody therapies, CGRP antibodies have the problem of being costly. However, taking into consideration the enormous socioeconomically burden that migraine is [87], the price may be well payed off.

In conclusion, based on current knowledge, we believe that the benefits of blocking CGRP – including the perspectives of improving the lives of those suffering from frequent headaches – seems to be greater than the disadvantages.
